# Glioblastoma Immunotherapy: A Systematic Review of the Present Strategies and Prospects for Advancements

**DOI:** 10.3390/ijms242015037

**Published:** 2023-10-10

**Authors:** Edoardo Agosti, Marco Zeppieri, Lucio De Maria, Camilla Tedeschi, Marco Maria Fontanella, Pier Paolo Panciani, Tamara Ius

**Affiliations:** 1 Department of Medical and Surgical Specialties, Division of Neurosurgery, Radiological Sciences and Public Health, University of Brescia, Piazza Spedali Civili 1, 25123 Brescia, Italy; edoardo_agosti@libero.it (E.A.);; 2 Department of Ophthalmology, University Hospital of Udine, P.le S. Maria della Misericordia 15, 33100 Udine, Italy; 3 Neurosurgery Unit, Head-Neck and NeuroScience Department, University Hospital of Udine, P.le S. Maria della Misericordia 15, 33100 Udine, Italy

**Keywords:** glioblastoma, immunotherapy, target therapies, vaccines, monoclonal antibodies, adoptive cell therapy, oncolytic viruses, systematic review, clinical outcomes

## Abstract

Glioblastoma (GBM) is characterized by aggressive growth and high rates of recurrence. Despite the advancements in conventional therapies, the prognosis for GBM patients remains poor. Immunotherapy has recently emerged as a potential treatment option. The aim of this systematic review is to assess the current strategies and future perspectives of the GBM immunotherapy strategies. A systematic search was conducted across major medical databases (PubMed, Embase, and Cochrane Library) up to 3 September 2023. The search strategy utilized relevant Medical Subject Heading (MeSH) terms and keywords related to “glioblastomas,” “immunotherapies,” and “treatment.” The studies included in this review consist of randomized controlled trials, non-randomized controlled trials, and cohort studies reporting on the use of immunotherapies for the treatment of gliomas in human subjects. A total of 1588 papers are initially identified. Eligibility is confirmed for 752 articles, while 655 are excluded for various reasons, including irrelevance to the research topic (627), insufficient method and results details (12), and being case-series or cohort studies (22), systematic literature reviews, or meta-analyses (3). All the studies within the systematic review were clinical trials spanning from 1995 to 2023, involving 6383 patients. Neuro-oncology published the most glioma immunotherapy-related clinical trials (15/97, 16%). Most studies were released between 2018 and 2022, averaging nine publications annually during this period. Adoptive cellular transfer chimeric antigen receptor (CAR) T cells were the primary focus in 11% of the studies, with immune checkpoint inhibitors (ICIs), oncolytic viruses (OVs), and cancer vaccines (CVs) comprising 26%, 12%, and 51%, respectively. Phase-I trials constituted the majority at 51%, while phase-III trials were only 7% of the total. Among these trials, 60% were single arm, 39% double arm, and one multi-arm. Immunotherapies were predominantly employed for recurrent GBM (55%). The review also revealed ongoing clinical trials, including 9 on ICIs, 7 on CVs, 10 on OVs, and 8 on CAR T cells, totaling 34 trials, with phase-I trials representing the majority at 53%, and only one in phase III. Overcoming immunotolerance, stimulating robust tumor antigen responses, and countering immunosuppressive microenvironment mechanisms are critical for curative GBM immunotherapy. Immune checkpoint inhibitors, such as PD-1 and CTLA-4 inhibitors, show promise, with the ongoing research aiming to enhance their effectiveness. Personalized cancer vaccines, especially targeting neoantigens, offer substantial potential. Oncolytic viruses exhibited dual mechanisms and a breakthrough status in the clinical trials. CAR T-cell therapy, engineered for specific antigen targeting, yields encouraging results, particularly against IL13 Rα2 and EGFRvIII. The development of second-generation CAR T cells with improved specificity exemplifies their adaptability.

## 1. Introduction

Glioblastoma (GBM) retains its status as the foremost prevalent and most malignant glial tumor. It accounts for over 50% of all primary brain tumors in the United States, presenting an annual incidence rate of approximately 3 cases per 100,000 individuals [1]. To date, GBM remains characterized by its high aggressiveness and unresponsiveness to nearly all current standard-of-care interventions, which encompass the combined use of chemotherapy and radiation post-surgical resection. Indeed, in spite of these aggressive treatment regimens, the median overall survival (OS) of afflicted patients extends no more than 15 months from their initial enrollment, just preceding the commencement of radiation therapy (RT) and concurrent chemotherapy (CT) involving temozolomide. This outcome is accompanied by a disheartening 5-year OS rate of less than 10%.

The difficulty of GBM treatment is primarily attributed to the limited population of therapy-resistant glioblastoma stem cells (GSCs) and the intricate tapestry of inter- and intra-tumor heterogeneity, which comprises various GBMs subtypes and stromal cells within the tumor microenvironment (TME) [2]. The GSCs bear significant responsibility for the recurrence of glioblastoma and its resistance to therapy, owing to their robust DNA repair mechanisms, [2] multi-drug-resistance traits, [3] and adept immune evasion tactics. The GBM TME plays a pivotal role in governing cellular behavior, driving GSC adaptability, and ultimately fostering therapeutic resistance [2,4,5]. Furthermore, GBM cells attract and modify immune cells distinct from microglia, reinforcing tumor growth and cultivating an immunosuppressive TME through the secretion of cytokines, extracellular vesicles, and the formation of connecting nanotubes [5].

The effectiveness of immunotherapy has already been firmly established across various solid tumor types, including melanoma, prostate cancer, non-small-cell lung cancer, and renal cell carcinoma. This success marks a notable advancement in the burgeoning field of immunotherapy, which hinges on the concept of re-educating and harnessing the patient’s immune response to combat tumors. These methods have progressively found their place in the treatment of a variety of cancers, including those afflicting the brain. Contemporary strategies for immunotherapy in cancer treatment primarily revolve around immune checkpoint blockade (ICB) agents. Furthermore, therapeutic vaccines, adoptive cell therapy, monoclonal antibodies (mAbs), and oncolytic viruses also constitute essential components of current immunotherapeutic approaches [1,4,6,7,8,9,10,11,12].

Numerous clinical trials have explored the application of immunotherapy in the context of GBMs. Nevertheless, due to the wide array of immunotherapy approaches employed, the varying selection of molecular targets, and the diversity of combination therapy strategies, questions persist concerning the effectiveness and safety of immunotherapies for GBM. Additionally, there is a noticeable absence of a recent comprehensive systematic review addressing the current strategies and future prospects of GBM immunotherapy [1,6,7].

This systematic literature review comprehensively examines a range of prominent immunotherapeutic strategies aimed at combating GBMs, including adoptive cellular transfer chimeric antigen receptor (CAR) T and NK cells, oncolytic viruses (OVs), cancer vaccines (CVs), immune checkpoint inhibitors (ICIs), and multifaceted combination therapies. Additionally, we provide a brief discussion on the rationale underpinning these approaches, while shedding light on their inherent limitations and the unique challenges that arise during the treatment of GBMs.

## 2. Methods

### 2.1. Literature Review

The systematic review was performed following the Preferred Reporting Items for Systematic Reviews and Meta-Analysis (PRISMA) guidelines [12]. Two authors performed a systematically comprehensive literature search of the PubMed, Ovid MEDLINE, and Ovid EMBASE databases. The first literature search was performed on 10 August 2023, and the search was updated on 3 September 2023. A combination of keyword searches was performed to generate a search strategy. The search keywords, including “glioblastoma”, “immunotherapy”, “target therapy”, “vaccines”, “monoclonal antibodies”, “overall survival”, and “progression free survival”, were used in both AND and OR combinations. Studies were retrieved using the following Medical Subject Heading (MeSH) terms and Boolean operators: (“glioma” OR “glioblastoma” OR “GBM”) AND (“immunotherapy” OR “target therapy”) AND (“outcomes” OR “prognosis” OR “progression free survival” OR “overall survival”). Other pertinent articles were identified through reference analysis of selected papers. A search filter was set to show only publications over the designated period: 1990–2023.

All studies were selected based on the following inclusion criteria: (1) English language; (2) clinical trials, including: single-arm or double-arm studies, and among them randomized controlled or non-randomized controlled trials; (3) studies on GBM immunotherapy strategies, both as stand-alone and combined therapies with CT and/or RT; and (4) studies including at least OS and progression free survival (PFS) among the outcomes analyzed. The following exclusion criteria were employed: (1) editorials, case reports, case series, cohort studies, literature reviews, and meta-analyses; (2) studies that did not clearly define the methods and/or results; and (3) studies that did not report data on PFS or OS.

The list of identified studies was imported into Endnote X9 and duplicates were removed. Two independent researchers (E.A. and P.P.P.) checked the results according to the inclusion and exclusion criteria. A third reviewer (M.Z.) resolved all disagreements. Then, the eligible articles were subject to full-text screening.

### 2.2. Data Extraction

For each study, we abstracted the following information: authors, year and journal of publication, title, name and phase of the clinical trial, number of patients, diagnosis, follow-up length, immunotherapy treatment, and outcomes.

### 2.3. Outcomes

Our primary outcomes were OS and PFS related to GBM immunotherapy.

### 2.4. Risk of Bias Assessment

**Figure 1:**
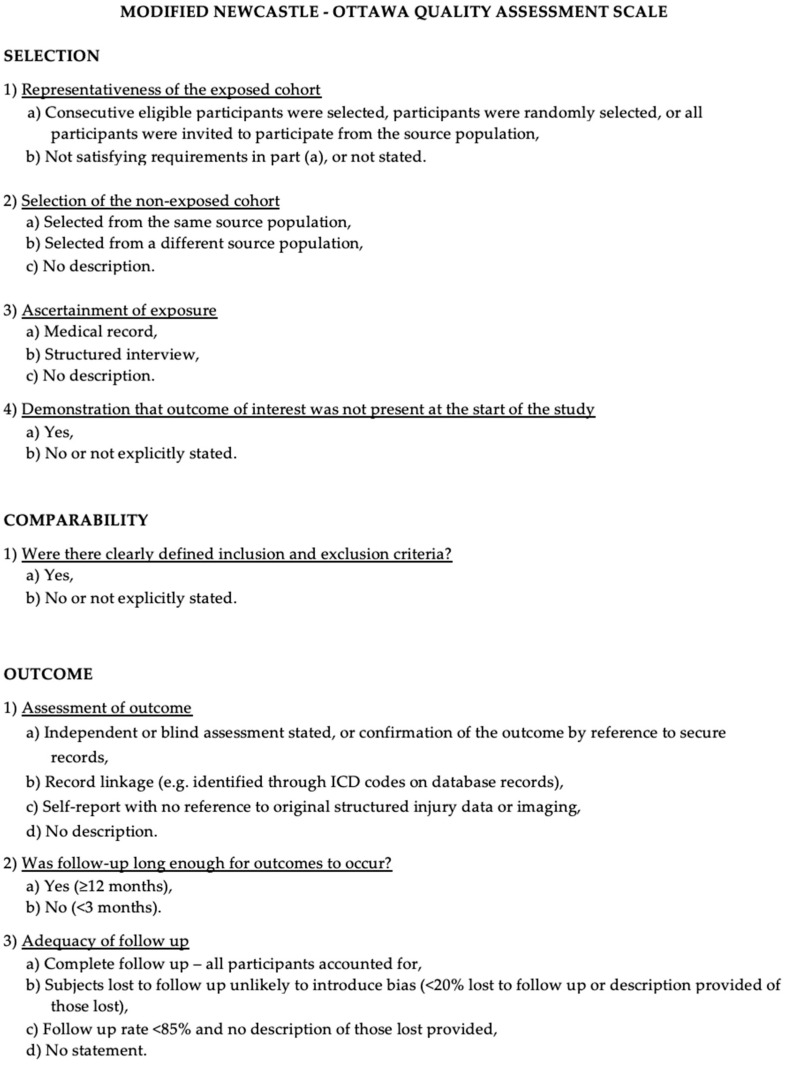
Modified Newcastle–Ottawa scale.

The Newcastle–Ottawa scale (NOS) [13] was used to assess the quality of the included studies. A quality assessment was performed by assessing the selection criteria, comparability of the study, and outcome assessment. The ideal score was 9. Higher scores indicated a better quality of studies. Studies receiving 7 or more points were considered high-quality studies. Two authors (E.A. and P.P.P.) performed the quality assessment independently. When discrepancies arose, the papers were re-examined by the third author (Figure 1).

### 2.5. Statistical Analysis

Descriptive statistics were reported, including ranges and percentages. All statistical analyses were performed using the R statistical package v3.4.1 http://www.r-project.org (accessed on 6 September 2023).

## 3. Results and Discussion

### 3.1. Literature Review Results

**Figure 2:**
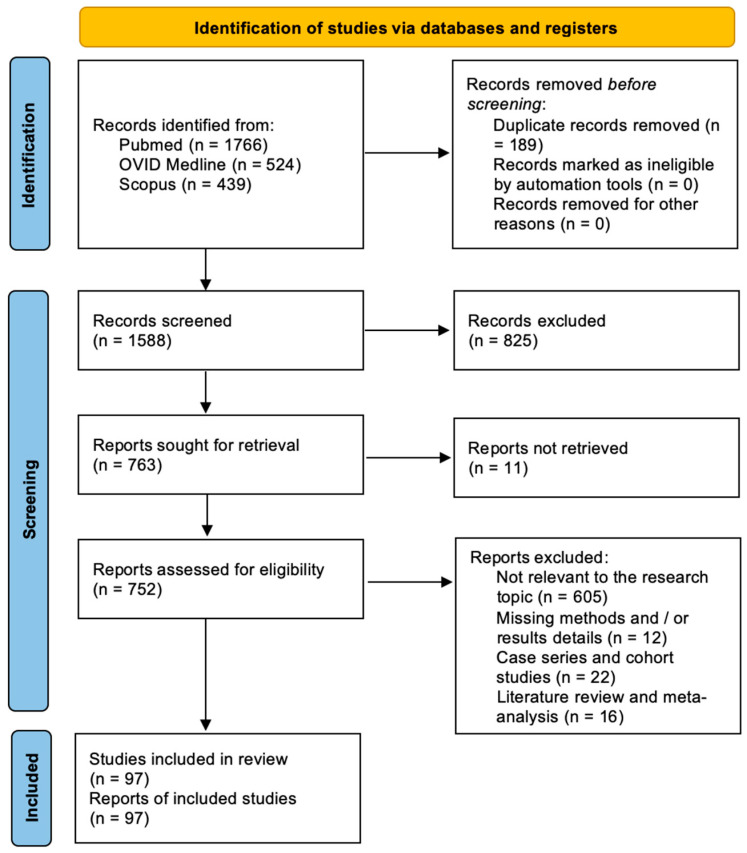
PRISMA flowchart.

A total of 1588 papers were identified after duplicate removal. After title and abstract analyses, 763 articles were identified for a full-text analysis. Eligibility was ascertained for 752 articles. The remaining 655 articles were excluded for the following reasons: (1) not relevant to the research topic (627 articles), (2) lack of method and/or results details (12 articles), case series and cohort studies (22 articles), and systematic literature review or meta-analysis (3 articles). All studies included in the analysis had at least one or more outcome measures available for one or more of the patient groups analyzed. Figure 2 shows the flowchart according to the PRISMA statement.

**Figure A1:**
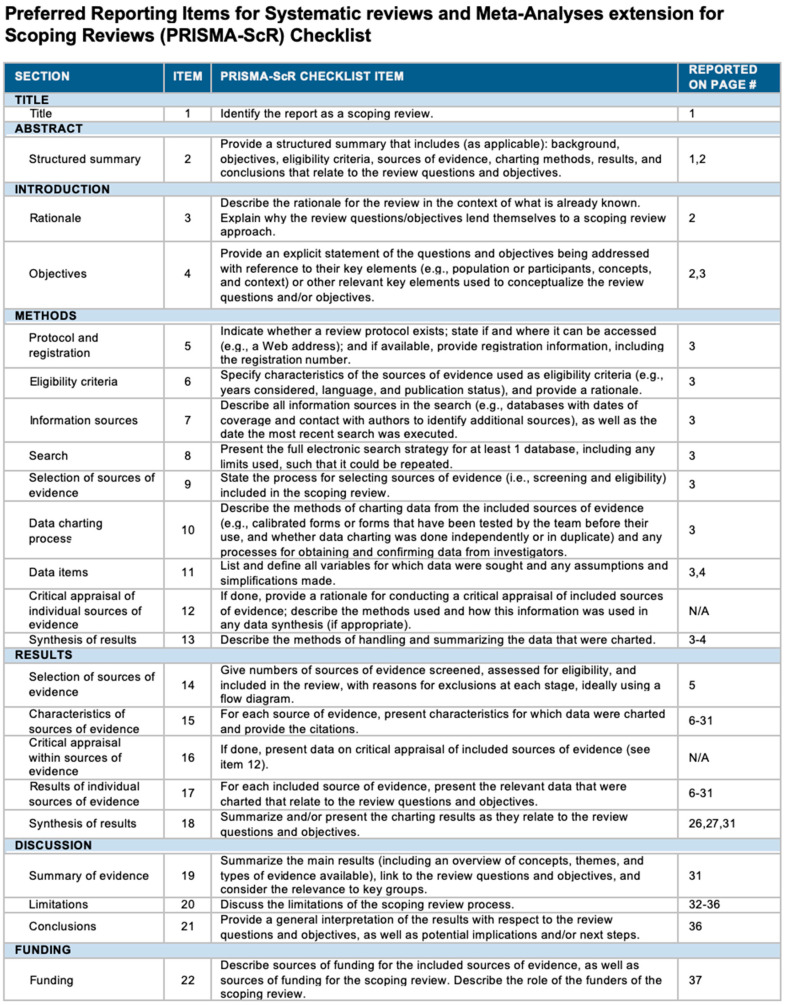
Preferred Reporting Items for Systematic Reviews and Meta-Analyses extension for Scoping Review (PRISMA-ScR) checklist.

The PRISMA checklist is available as Appendix A (Figure A1).

### 3.2. Data Analysis

A summary of the included studies is presented in Table 1.

All the studies included in our systematic review were clinical trials, with study periods ranging from 1995 to 2023. In total, 6383 patients were enrolled in these trials. *Neuro-oncology* is the scientific journal that has published the highest number of clinical trials related to glioma immunotherapy (15/97, 16%), followed by *Clinical Cancer Research* (9/97, 9%), and *Cancer Immunology Immunotherapy* (6/97, 6%). The majority of these studies were published between 2018 and 2022, with 9 studies published each year during this period.

In terms of the classes of immunotherapeutic agents studied, least of the research focused on CAR T cells, accounting for 11/97 (11%) of the studies. Other significant categories included ICIs at 25/97 (26%), OVs at 12/97 (12%), and CVs at 49/97 (51%). Among these studies, phase-I trials were the most numerous, comprising 51% (49”97), while only 7% (7/97) were phase-III trials. Of the clinical trials, 60% (60/97) were single-arm studies, 39% (38/97) were double-arm studies, and only 1 was a multi-arm study. Among the double-arm and multi-arm trials, 85% (33/39) were randomized controlled trials. Among these, 61% (20/33) demonstrated improved OS and/or PFS compared to conventional treatment.

Immunotherapies were predominantly used for recurrent GBM in 55% of cases (53/97), while in the remaining cases, they were used as the initial treatment. In 25% of the latter cases (11/44), immunotherapies were used as the sole therapy without the addition of other adjuvant therapies, such as RT and/or CT.

A summary of the ongoing studies on GBM immunotherapies is presented in Table 2, Table 3, Table 4 and Table 5.

From the review, it emerged that 9 clinical trials were ongoing on ICIs, 7 on CVs, 10 on OVs, and 8 on CAR T cells, for a total of 34 clinical trials. Of these, most (18/34, 53%) were phase-I clinical trials, while only one was phase III.

### 3.3. Discussion

In this study, we conducted a systematic review of all the clinical trials published and ongoing between 1995 and 2023. Our findings reveal that the primary classes of immunotherapeutic agents under investigation include CAR T cells (11%), ICIs (26%), OVs (12%), and CVs (51%). Phase-I trials constituted the majority, accounting for 51% of the studies, whereas phase-III trials comprised only 7%. Double-arm studies constituted only 39%; however, among them, 85% were randomized controlled trials. Among these trials, 61% demonstrated improved overall survival (OS) and/or progression free survival (PFS) rates when utilizing GBM immunotherapy, both as a standalone strategy and in combination with conventional treatment. A total of 34 clinical trials are ongoing and the majority of them are phase-I CTs.

#### 3.3.1. Immunosuppressive Mechanisms Employed by GBM

Glioblastoma, recognized as the most aggressive primary brain tumor, poses significant challenges due to its rapid growth, capacity to infiltrate brain tissue, molecular diversity, and resistance to treatment [110]. While immunotherapy holds promise in combating GBM, it encounters formidable obstacles related to the immunological environment of the central nervous system (CNS). Glioblastoma exhibits traits typical of immunotherapy-responsive tumors but deploys extensive immunosuppressive mechanisms, leveraging its CNS location. Overcoming intrinsic resistance, countering systemic immunosuppression, addressing adaptive resistance, and adapting to acquired resistance are crucial to dismantle GBM’s immunosuppressive machinery [3,111,112,113,114,115].

Intrinsic resistance in GBM is influenced by its molecular and clinical characteristics. Studies have revealed intratumor heterogeneity, where different subtypes coexist within the same tumor, complicating selective eradication of treatment-susceptible clones and paving the way for resistant ones. To navigate this complexity, immunotherapy aims to target multiple neoantigens derived from autologous tumor cells, minimizing the risk of antigenic overlap with normal tissue [113].

Immunotherapy’s success hinges on targeting neoantigens essential for tumor survival across GBM subtypes while sparing healthy tissue. Various approaches, such as OVs, CVs, and ICIs, address neoantigens effectively. However, CAR T- and NK-cell therapies require alternative strategies. The GBM microenvironment significantly contributes to immune evasion through immunosuppression. Microglia, the primary antigen-presenting cells in the CNS, promote tumor infiltration, progression, and invasiveness. Inflammatory cytokines secreted by microglia amplify GBM expansion. Moreover, the blood–brain barrier (BBB) typically prevents immune cell entry; however, GBM cells release chemotactic signals, such as CCL2, CCL5, CXCL, and SDF-1, to actively recruit tumor-associated macrophages (TAMs) across the BBB. TAMs in GBM are known to enhance immune checkpoints and support cancer stem cells. Inhibiting the activity of CCL2 and CCL5 has shown promise in reducing tumor migration and invasion [116,117].

#### 3.3.2. Immunotherapeutic Strategies

In pursuit of eradicating GBM cells, particularly the therapy-resistant subset, immunotherapy aims to activate the patient’s antitumor immune response. Our review reveals a variety of immunotherapeutic strategies, such as CVs, OVs, ICIs, and CAR T cells, which are evaluated in clinical studies either individually or in conjunction with standard GBM treatments.

##### Immune Checkpoint Inhibitors

Immune checkpoint inhibitors are monoclonal antibodies designed to counteract negative regulatory pathways that impede T-cell activation. These antibodies target surface receptors known as immune checkpoints. Under normal circumstances, immune checkpoint molecules can dampen cytotoxic T-cell function. However, when ICIs are employed, they disrupt this normal activation of immune checkpoints, restoring T-cell function and enhancing the immunotherapeutic effect. Presently, the primary focus of immune checkpoint inhibition revolves around two key receptors: programmed cell death protein 1 (PD-1) and cytotoxic T-lymphocyte-associated protein 4 (CTLA-4). Notable advancements have been made in treating challenging cancers, such as melanoma, lung cancer, and renal cancer, using anti-PD-1 and anti-PD-L1 antibodies [75,93,118,119,120,121,122].

The NCT02017717 trial, a pioneering randomized phase-I clinical study for recurrent GBM, assessed the efficacy and tolerability of nivolumab (a PD-1 inhibitor) as monotherapy or in combination with ipilimumab (a CTLA-4 inhibitor). All patients underwent surgical resections, RT, and TMZ treatments before being allocated to three different treatment arms. Intriguingly, nivolumab monotherapy demonstrated a superior median overall survival (10.4 months) compared to the NIVO1 + IPI3 or NIVO3 + IPI1 combinations (9.2 and 7.3 months, respectively). Phase III of this trial involved 369 recurrent GBM patients randomized to receive either nivolumab or bevacizumab. While mOS and toxicity were comparable, bevacizumab-treated patients exhibited a shorter duration of radiologic responses [118].

A phase-II clinical trial assessed bevacizumab alone or combined with notecan for efficacy in recurrent GBM patients, with both treatment arms showing tolerable side effects and median overall survival rates of 9.2 and 8.7 months, respectively.

At present, two ongoing phase-III trials, NCT02667587 and NCT02617589, are investigating nivolumab’s potential as a treatment for MGMT-unmethylated glioblastoma. The NCT02667587 clinical trial compares SOC with nivolumab or placebo, while NCT02617589 compares nivolumab versus TMZ, each in combination with RT.

Pembrolizumab, another anti-PD1 checkpoint inhibitor, is being investigated as a glioma treatment. A phase-II trial involving neoadjuvant pembrolizumab administration before surgery, followed by post-surgery adjuvant treatment, demonstrated increased survival in recurrent GBM patients.

CTLA-4, an immune checkpoint that competes with CD80 and CD86 for binding, thereby suppressing T-cell function, is under evaluation in clinical trials (NCT02311920, NCT02829931) using ipilimumab therapies.

Another immune checkpoint receptor, LAG-3, inhibits T-cell activity while enhancing the suppressive function of Tregs. A phase-I trial (NCT02658981) is currently assessing BMS-986,016, a LAG-3 inhibitor, alone and in combination with nivolumab in recurrent GBM patients. TIM-3, an additional lymphocyte-expressed receptor capable of inducing T-cell exhaustion and immune response suppression, can lead to unfavorable outcomes [4,92,118,119,120,121,122].

##### Cancer Vaccines

Vaccines hold promise as tumor treatment, leveraging tumor antigens. They activate immune surveillance against GBM, fortifying the adaptive immune system. GBM vaccines fall into four categories: peptides, DNA, cells, and mRNA. Peptide and DNA vaccines administer tumor-specific antigens or DNA to provoke an adaptive immune response. Cell vaccines, specifically DC vaccines derived from PBMCs, prime with tumor antigens. mRNA vaccines employ viral vectors loaded with mRNA, generating potent immune responses. Despite years of development, vaccines remain under investigation. Of these, only three have advanced to phase-III clinical trials: Rindopepimut, DCvax, and PPV.

Rindopepimut, a peptide-based vaccine, targets EGFRvIII mutation exclusively in GBM, minimizing off-tumor toxicity risk. However, GBM’s tumor heterogeneity poses challenges, as EGFRvIII expression varies. Phase-II trials demonstrated improved progression free survival (PFS) and median survival, compared to historical controls. ACT IV, a double-blind phase-III trial, examined Rindopepimut’s effect on GBM patients with minimal residual disease (MRD). MRD was defined as <2 cm^2^ of enhancing tumor tissue post-surgery and chemoradiotherapy. The trial showed no significant difference in overall survival for MRD patients. IDH1 peptide vaccines, targeting the IDH1R132 H mutation, are in phase-I trials following promising preclinical results. Survivin, highly expressed in GBM, led to the development of SurVaxM, a peptide vaccine. Early results from a phase-II study combining SurVaxM with TMZ show improved PFS and OS. An antisense oligodeoxynucleotide against the IGF type-I receptor (IMV-001) demonstrated improved PFS in phase-I trials. PPV immunotherapy displayed safety and efficacy in a phase-I trial for recurrent GBM patients. However, a phase-III trial comparing PPV to best supportive care yielded unfavorable results. Neoantigens, identified through DNA and RNA sequencing, offer a personalized approach to GBM treatment. Two clinical trials demonstrated the potential of personalized GBM vaccination.

In the GAPVAC study, 16 newly diagnosed GBM patients received two synthesized vaccines: one targeting unmutated peptides (APVAC1) and the other neoantigens (APVAC2). Both vaccines elicited CD4+ and CD8+ T-cell responses, with varying immunogenicity. APVAC1 induced 50% immunogenicity, while APVAC2 achieved 84.7%. APVAC1 primarily stimulated CD8+ T-cell responses, while APVAC2 focused on CD4+ T-cell responses. Median PFS and OS were encouraging.

Another study administered a neoantigen vaccine (NeoVax) to newly diagnosed and MGMT unmethylated GBM patients. Two patients displayed immunogenicity and both CD4+ and CD8+ T-cell responses. Median PFS reached 7.6 months, with a median OS of 16.8 months. DCVax-L, a personalized peptide vaccination, demonstrated potential in a phase-III trial when combined with standard GBM therapy. mRNA-transfected DC vaccines were well-tolerated and significantly extended PFS compared to controls in a phase-II trial. Autologous DCs, pulsed with autologous whole tumor lysate and combined with standard chemoradiotherapy, proved feasible and safe in newly diagnosed GBM patients. In contrast, a multicentric phase-II study involving tumor lysate-charged autologous DCs (Audencel) failed to improve clinical outcomes for newly diagnosed GBM patients. AV-GBM-1, autologous DCs loaded with tumor-associated antigens from short-term autologous tumor cell cultures, is being evaluated in an ongoing phase-II clinical trial.

Dendritic cells (DCs), capable of promoting adaptive antitumor immune responses, are ideal for cellular vaccination. DC vaccines demonstrated effectiveness in preclinical and early stage clinical trials, showing significantly longer overall survival in GBM patients. Intratumoral and intradermal administrations yielded better results than intradermal alone. ICT-107, a DC-based vaccine designed for newly diagnosed GBM patients, proved safe with encouraging median PFS and OS in phase-I trials. A phase-III trial comparing ICT-107 to standard care was suspended due to funding issues.

The landscape of GBM treatment is evolving, with vaccines offering a promising avenue for intervention. While many vaccine candidates are in development, only a select few have progressed to phase-III trials. The complex nature of GBM, including its heterogeneity, presents both challenges and opportunities in vaccine development. Neoantigens, identified through advanced sequencing techniques, offer a personalized approach that shows significant potential. Additionally, DC-based vaccines have demonstrated effectiveness in the preclinical models and early stage clinical trials, with some promising results for patients. The ongoing quest for effective GBM vaccines continues, with researchers exploring various strategies to harness the power of the immune system in the fight against this devastating disease [24,46,53,55,56,64,123,124,125,126,127,128,129,130,131,132,133].

##### Oncolytic Viruses

In recent years, OVs have emerged as a novel therapeutic approach in treating various solid tumors, including GBM. OVs offer a dual mechanism of antitumor action, involving the direct killing of tumor-specific cells and the induction of systemic antitumor immunity, encompassing both innate and adaptive responses. OVs trigger immunogenic cell death in tumor cells, leading to the release of tumor-associated antigens (TAAs), damage-associated molecular patterns (DAMPs), and pathogen-associated molecular patterns (PAMPs). DAMPs and PAMPs serve as potent stimulators of innate immunity by activating pattern recognition receptors, such as Toll-like receptors. Furthermore, these molecules enhance antigen cross-presentation and adaptive immune responses.

OVs also elicit a proinflammatory immune response, increasing the production of CXCL9, CXCL10, and CXCL11, which promote the trafficking and infiltration of T cells into tumors. Various OVs, including adenovirus, herpes simplex virus, measles virus, parvovirus, poliovirus, and zika virus, have demonstrated efficacy against GBM in preclinical studies. Encouraging data from clinical trials have shown OVs to have a favorable safety profile and promising efficacy, with evidence of intratumoral viral replication and lymphocyte infiltration. For instance, a phase-I clinical trial (NCT01470794) demonstrated the safety and efficacy of Toca 511 in treating 56 recurrent high-grade glioma patients. In a follow-up phase-III study involving 23 eligible patients, the median overall survival (OS) reached 14.4 months, with one- and two-year survival rates of 65.2% and 34.8%, respectively. Notably, five patients achieved complete responses and survived for extended periods after Toca 511 treatment.

DNX-2401, a tumor-selective oncolytic adenovirus, exhibited antiglioma efficacy in preclinical studies. In a phase-I dose-escalation trial involving 37 patients with recurrent malignant gliomas, DNX-2401 demonstrated both safety and promising responses across different dose levels. Approximately 20% of patients survived beyond three years post-treatment, and some patients experienced over a 95% reduction in tumor size with a progression free survival exceeding three years. Analyses of post-treatment tumor specimens revealed viral replication and spread within the tumor, along with the induction of intratumoral CD8+ and T-bet+ T-cell infiltration and a reduced expression of transmembrane immunoglobulin mucin-3.

Both Toca 511 and DNX-2401 trials reported that approximately 20% of GBM patients exhibited complete responses after intratumoral OV administration, with rare virotherapy-associated severe adverse events. Oncolytic H-1 parvovirus, although showing only a slight improvement in the median OS when administered intratumorally to GBM patients, displayed an increase in infiltrating lymphocytes and IFN-γ levels.

PVSRIPO, a live attenuated poliovirus type-1 vaccine with a modified internal ribosome entry site, received a breakthrough therapy designation from the FDA based on a phase-I study in recurrent GBM patients. PVSRIPO recognizes the poliovirus receptor CD155, which is upregulated on malignant and antigen-presenting cells within the tumor microenvironment. The phase-I study demonstrated safety and sustained survival rates, with about 20% of patients remaining alive for 57–70 months after PVSRIPO injection. A phase-II randomized trial of PVSRIPO alone or in combination with lomustine in patients with recurrent grade-IV malignant glioma (NCT02986178) is ongoing.

Herpes simplex virus-1 (HSV-1), a double-stranded DNA virus, has been extensively explored as a treatment for various solid tumors, including GBM. Genetically engineered variants, such as G207 and G47 delta, have shown promise. In a phase-I clinical trial involving children and adolescents with recurrent or progressive high-grade gliomas, G207 demonstrated safety and enhance the immunological response.

G47 delta (DELYTACT), another oncolytic HSV-1 variant, was evaluated in adult patients with residual or recurrent GBM in a single-arm phase-II clinical trial in Japan. Minimal side effects were observed, and the survival rate after one year reached an impressive 84.4%. G47 delta (Delytact/Teserpaturev) received conditional approval from the Japan Ministry of Health, Labor and Welfare (MHLW) for the treatment of malignant gliomas in Japan [49,54,55,56,66,68,134,135].

Several ongoing clinical trials are exploring the use of OVs as therapeutic agents for recurrent high-grade gliomas. While these results are promising, further clinical trials are needed to establish the safety and efficacy of OVs as a therapy for GBM.

##### CAR T Cell

CAR T-cell therapy presents a promising strategy for overcoming the formidable challenges posed by the BBB and the intricate tumor microenvironment (TME) within the context of adoptive cell T-cell therapy (ACT). ACT involves the reintroduction of autologous or allogenic anti-tumor T cells engineered to target tumor-specific antigens highly expressed on tumor cells while sparing normal cells. In a meticulously orchestrated process, these T cells are meticulously engineered in vitro using a lentiviral vector, thereby expressing a high-affinity single-chain fragment variable (scFv) tailored to the target antigen. This scFv is intricately fused with transmembrane regions, co-stimulatory domains, and an intracellular signaling domain derived from the CD3 molecule of the T-cell receptor (TCR). This engineering empowers autologous T cells to keenly recognize antigens upon scFv binding, initiating CAR molecule clustering and ensuing activation. This activation, in turn, triggers a cascade of events, including cytokine release, proliferation, cytotoxicity, and metabolic shifts, thereby enabling CAR T cells to chiefly exert their anti-tumor functions through cytokine release and the involvement of the granzyme and perforin axis, as well as the Fas and Fas ligand axis, thus equipping them to effectively surmount TME-related immunosuppression.

Notably, CAR T cells meticulously engineered to target IL13 Rα2 have demonstrated a heightened degree of selectivity for this receptor over IL13Ra1/IL4Ra. Intracranial injections of these IL13-zetakine CAR T cells, executed within a glioma xenograft model, have yielded auspicious improvements in median overall survival. A groundbreaking first-in-human pilot safety and feasibility trial conducted in 2015 evaluated the application of IL13-zetakine CAR T cells in three patients with recurrent GBM. These CAR T cells were methodically delivered via an implanted reservoir/catheter system, directly into the resection cavity, thereby resulting in a precisely controlled, transient bout of brain inflammation. Although the CAR T cells were met with overall favorable tolerability, certain adverse events, including grade-3 headaches and transient grade-3 neurologic events, were observed. A subsequent analysis of tumor tissue indicated a tangible reduction in IL13 Rα2 expression, while an MRI analysis suggested an increase in tumor necrotic volume, thereby potentially extending overall survival. Following this pioneering trial, a second-generation IL13-zetakine CAR T cell was painstakingly developed, featuring a 4-1BB costimulatory domain and an ingeniously mutated IgG4-Fc linker, all designed to amplify antitumor potential while curbing off-target interactions. A patient afflicted with recurrent GBM subsequently received intracavitary infusions of these CAR T cells, resulting in a pronounced inhibition of local tumor progression. However, the emergence of novel intracranial tumors and spinal lesions was noted, with the fifth intraventricular infusion precipitating a substantial reduction in tumor size. Regrettably, this regression proved transient, lasting merely 7.5 months, and recurrence subsequently manifested at new locations, characterized by diminished IL13 Rα2 expression, thereby underscoring the indispensable need for combinatory therapeutic modalities aimed at addressing antigen-loss relapse.

EGFRvIII, a frequently encountered GBM mutation, was initially associated with abbreviated survival periods. In a phase-I clinical trial, 18 patients afflicted with recurrent GBM received third-generation EGFRvIII-directed CAR T cells, endowed with CD28 and 4-1BB costimulatory domains. Patients, upon infusion of these CAR T cells, expeditiously developed respiratory symptoms, particularly evident at higher dose levels, a phenomenon indicative of dose-limiting toxicity, despite the absence of discernible clinical benefits. The median overall survival rate reached 6.9 months, with select patients achieving survival exceeding a year. To effectively address instances of EGFRvIII-negative yet EGFR-positive glioblastomas, a novel approach surfaced, involving the fusion of EGFRvIII scFv with a bispecific T-cell engager (BiTE). These BiTEs, as synthetic bispecific antibodies, serve to heighten immune interactions while simultaneously augmenting antibody specificity. Recent investigations have explored SynNotch-CAR T cells, meticulously designed to target multiple antigens, thus yielding improvements in specificity, comprehensiveness, and longevity in comparison to conventional T-cell therapy, as employed in the context of glioblastoma.

B7-H3, alternatively known as CD276, plays a pivotal role in regulating T-cell functions and, notably, is found to be overexpressed across a diverse array of human cancer cells, a correlation that frequently coincides with negative clinical outcomes. This distinct profile renders it an enticing target for immunotherapeutic interventions. Nevertheless, significant challenges persist, not the least of which is the full elucidation of its receptor and the intricacies of immune regulation associated therewith.

HER2, conspicuously expressed in various CNS tumors, including GBM, yet conspicuously absent in normal CNS tissue, emerges as an attractive and distinctively selective target. Clinical trials employing HER2-specific CAR T cells have borne witness to well-tolerated treatments, despite the lack of substantial and tangible survival benefits. Recent preclinical inquiries have embarked upon the exploration of trivalent CAR T-cell therapy, an innovative approach that targets HER2, IL13 Rα2, and ephrin-A2, demonstrating encouraging improvements in terms of cytokine release and cytotoxicity when contrasted with the outcomes achieved using monospecific or bispecific CAR T cells, thereby strongly hinting at the potential utility of this multifaceted therapeutic approach within the context of glioblastoma therapy.

CAR T-cell therapy, undoubtedly, occupies a paramount and promising position in the realm of glioblastoma immunotherapy. Recent strides and advancements, such as the development of second-generation CAR T cells with heightened specificity and reduced off-target effects, serve as a testament to the adaptability and burgeoning potential of this therapeutic modality. Strikingly promising results have emerged from studies targeting antigens, such as IL13 Rα2 and EGFRvIII, as reflected in the significant tumor regression and improved patient survival rates. Moreover, the visionary concept of trivalent CAR T cells augurs a future where combination therapies that adeptly target multiple antigens may yield even more favorable treatment outcomes. Though formidable challenges persist, encompassing the phenomena of antigen loss and the imperative [74,136,137,138,139,140,141,142,143,144,145,146,147,148,149].

## 4. Conclusions

Our comprehensive analysis of the immunotherapy strategies for GBM treatment highlights the evolving landscape of therapeutic interventions for this challenging disease. A diverse range of approaches, including CAR T cells, ICIs, OVs, and CVs, are being explored to address the unique challenges presented by GBM. To develop a curative immunotherapy for GBM, it is crucial to overcome immunotolerance, stimulate robust responses to tumor antigens, and effectively counter the evolving escape mechanisms within the immunosuppressive microenvironment.

Immune checkpoint inhibitors, such as PD-1 and CTLA-4 inhibitors, have demonstrated promise in the clinical trials, and the ongoing research continues to seek ways to enhance their effectiveness. Cancer vaccines, particularly those targeting neoantigens, offer a personalized approach with significant potential. Oncolytic viruses have shown dual mechanisms of action and have displayed promise in clinical trials, with some achieving breakthrough status. CAR T-cell therapy shines as a beacon of hope. These cells, intricately engineered to target specific antigens, have produced encouraging results, especially when targeting IL13 Rα2 and EGFRvIII. The development of second-generation CAR T cells with improved specificity and reduced off-target effects exemplifies the adaptability and potential of this approach.

Despite this promising data from published and ongoing studies on GBM immunotherapies, the considerable variability in these results and the multitude of therapeutic targets make it challenging to reach consensus. Therefore, translating these results into clinical success for GBM patients remains a formidable task. Nevertheless, ongoing studies are actively exploring combination approaches and enhancing existing immunotherapeutic strategies, offering hope for a revolutionary breakthrough in cancer care.

## Figures and Tables

**Table 1:** Summary of the studies included in the systematic literature review.

Author	Year	Trial Name	Phase	Patients (N)	Diagnosis (Target Glioma)	Follow Up (Months, Median Value)	Treatment	Endpoints	
								OS	PFS
Riva et al. [14]	1994	N/A	I	24	Recurrent HGG	N/A	Adjuvant RIT: murine monoclonal anti-Tenascin Ab (BC-2 and BC-4) labeled with 131I injected directly into the tumor through a catheter	mOS: 16 mo	N/A
Riva et al. [15]	1995	N/A	I	50	GBM	N/A	Adjuvant RIT: murine monoclonal anti-Tenascin Ab (BC-2 and BC-4) labeled with 131I locally infused in the site of neoplastic disease through a catheter + RT and CHT	mOS: 20 mo	N/A
Pöpperl et al. [16]	2002	N/A	I	12	GBM	N/A	Adjuvant RIT: murine monoclonal anti-Tenascin Ab (BC-2 and BC-4) labeled with 131I locally infused in the surgical cavity (compared with an historical control group (n = 85) treated with standard therapy)	mOS: 18.5, mOS in historical control group: 9.7 mo	N/A
Fukushima et al. [17]	2003	N/A	I	26	GBM	N/A	RT + MCNU (ranimustine) + TNF-SAM2 (recombinant human mutant TNF-α) (compared with an historical control group (n = 26) treated with standard therapy)	mOS: 330 wk	N/A
Yu et al. [18]	2004	N/A	I	14	GBM	N/A	Autologous tumor lysate-pulsed DCV	mOS: 133 wk	N/A
Steiner et al. [19]	2004	N/A	II	10	GBM	49	(1) ATV-NDV (NDV-modified autologous tumor vaccine) + SOC (2) Standard therapy	(1) mOS: 100 wk, OS-1 y/2 y/3 y: 91%/39%/4% (2) mOS: 49 wk, OS-1 y/2 y/3 y: 45%/11%/0%	(1) mPFS: 40 wk, PFS-1 y/2 y: 21%/4% (2) mPFS: 26 wk, PFS-1 y/2 y: 8%/1%
Yamanaka et al. [20]	2005	N/A	I/II	35	Recurrent GBM	24	(1) Autologous tumor lysate-pulsed DCV + KLH or KLH/OK-432 (2) EBRT + nitrosourea-based CHT	(1) OS-2 y: 23.5% (2) OS-2 y: 3.7%	N/A
Vleeschouwer et al. [21]	2008	N/A	I	56	Recurrent GBM	16	Adjuvant autologous resected GBM lysate-pulsed (mature) DCV	mOS: 9.6 mo OS-12 mo/24 mo/36 mo: 37.4%/14.8%/11.1%	mPFS: 3 mo, PFS-12 mo: 10.7%
Izumoto et al. [22]	2008	N/A	II	21	Recurrent GBM	N/A	WT1-235 peptide vaccination	N/A	mPFS: 20 wk, PFS-6 mo: 33.3%
Ardon et al. [23]	2010	N/A	N/A	8	GBM	N/A	SOC + autologous GBM lysate-loaded DCV	N/A	PFS-6 mo: 75%, mOS: 24 mo
Sampson et al. [24]	2010	NCT00643097	II	35	GBM	N/A	(1) Newly diagnosed GBM EGFRvIII + PEPvIII-KLH (2) TMZ	N/A	(1) mPFS: 14.2 mo, mOS: 26 mo (2) mPFS: 6.3 mo, mOS: 15 mo
Okada et al. [25]	2011	N/A	I/II	22	Recurrent GBM	N/A	Vaccination with α-type 1 polarized DCs (αDC1) loaded with GAAs + immunoadjuvant poly-ICLC	N/A	PFS-12 mo: 41%
Prins et al. [26]	2011	NCT00068510	I	23	GBM	N/A	Autologous tumor lysate-pulsed DCV + Imiquimod or Poly-ICLC adjuvant	mOS: 31.4 mo, OS-1 y/2 y/3 y: 91%/55%/47%	N/A
Akiyama et al. [27]	2012	UMIN000000914	I	9	GBM	N/A	5 synthetic peptides-pulsed DCV + KLH (compared with an historical control group treated with standard therapy)	mOS 19 mo, mOS 16 mo of the historical control group	N/A
Valle et al. [28]	2012	N/A	I	5	GBM	N/A	Autologous tumor lysate-pulsed DCV	mOS: 27 mo, 2 y-OS: 80%	mPFS: 16.1 mo
Cho et al. [29]	2012	N/A	II	34	Newly diagnosed GBM	33	(1) SOC + adjuvant autologous tumor lysate-pulsed DCV (2) SOC (surgery + RT + CHT)	(1) mOS: 31.9 mo OS-1 y/2 y/3 y: 88.8%/44.4%/16.7% (2) mOS: 15 mo, OS-1 y/2 y/3 y: 75%/18.8%/0%	(1) mPFS: 8.5 mo (2) mPFS: 8 mo
Crane et al. [30]	2013	NCT00293423	I	12	Recurrent GBM	N/A	Adjuvant autologous HSPPC-96 vaccine	mOS in responder: 47 wk, mOS in non-responder: 16 wk	N/A
Phuphanich et al. [31]	2013	N/A	I	21	GBM	40	Autologous tumor lysate-pulsed DCV	mOS: 38.4 moOS-6 mo/12 mo/24 mo/36 mo: 100%/100%/93.7%/55.6%/38.4%	PFS-6 mo/12 mo/18 mo/24 mo: 100%/62.5%/43.8%/43.8%
Tanaka et al. [32]	2013	N/A	I	17	Recurrent GBM	N/A	Glutaraldehyde-fixed HUVEC vaccine (human umbilical vein endothelial cell)	OS-6 mo/1 y/5 y: 88.2%/47.1%/17.6%	mPFS: 5.5 mo
Pellegatta et al. [33]	2013	N/A	N/A	15	Recurrent GBM	N/A	Autologous tumor lysate-pulsed DCV	mOS: 8 mo	mPFS: 4.4 mo
Vik-Mo et al. [2]	2013	NCT00846456	I/II	17	GBM	N/A	(1) DCV with mRNA from GSC (2) without DCV	N/A	(1) mPFS: 694 d, mOS: 759 d (2) mPFS: 236 d, mOS: 585 d
Pollack et al. [34]	2014	NCT01130077	I	26	Pediatric BSG	N/A	Autologous tumor lysate-pulsed DCV	mOS in BSG patients: 12.7 mo; median OS in HGG patients: 25.1 mo	N/A
Schuessler et al. [35]	2014	ACTRN12609000338268	I	11	Recurrent GBM	N/A	ACT with CMV-specific autologous cytotoxic T cells	mOS: 403 d	mPFS: 246 d
Bloch et al. [36]	2014	NCT00293423	II	41	Recurrent GBM	N/A	Adjuvant autologous HSPPC-96 vaccine (peptide complexes bound to chaperon HSP-96, overexpressed in HGG)	OS-6 mo/12 mo: 90.2%/29.3%, mOS: 42.6 wk	N/A
Ishikawa et al. [37]	2014	UMIN000001426	I/II	24	Newly diagnosed GBM	30	Autologous formalin-fixed GBM tumor vaccine (AFTV) + FRT and TMZ	OS-2 y/3 y: 47%/38%, mOS: 22.2 mo	PFS-2 y: 33%, mPFS: 8.2 mo
Hashimoto et al. [38]	2015	N/A	I	7	GBM	N/A	WT1-peptide vaccination + TMZ	N/A	PFS: range 5.2–49-1 mo
Schijns et al. [39]	2015	N/A	I	48	Recurrent GBM	10	(1) Gliovac (or ERC 1671) vaccine (2) SOC	(1) OS-6 mo: 100%, OS-40 wk: 77% (2) OS-6 mo: 33%, OS-40 wk: 10%	N/A
Sakai et al. [40]	2015	N/A	I	10	Recurrent GBM	21	Autologous tumor lysate-pulsed DCV	mOS: 26 mo, OS-21 mo: 50%	N/A
Kalkanis et al. [41]	2015	NCT01156584	I	54	Recurrent HGG	N/A	(1) Toca 511 + Toca FC (2) External control	(1) mOS: 13.6 mo (2) mOS: 7.1 mo	N/A
Westphal et al. [42]	2015	N/A	III	142	Newly diagnosed GBM	N/A	(1) Nimotuzumab + SOC (2) SOC	(1) mOS: 19.5 mo (residual tumor) and 23.3 mo (no residual tumor) (2) mOS: 16,7 mo (residual tumor) and 21 mo (no residual tumor)	(1) PFS-12 mo: 25.6%, mPFS: 5.6 mo (residual tumor) and 10.6 mo (no residual tumor) (2) PFS-12 mo: 20.3% mPFS: 4 mo (residual tumor) and 9.9 mo (no residual tumor)
Akasaki et al. [43]	2016	N/A	I/II	32	Recurrent and newly diagnosed GBM	N/A	TMZ + immunotherapy with fusion cell (FC): autologous cultured GBM cell were fused with autologous DC using polyethylene glycol	Recurrent GBM: mOS: 18 mo; newly diagnosed GBM: mOS: 30.5 mo	Recurrent GBM: PFS: 10.3 mo; newly diagnosed GBM: mPFS: 18.3 mo
Brown et al. [44]	2016	NCT02208362	I	92	Recurrent GBM	N/A	IL13 Rα2-specific CAR T cells	N/A	PFS: 7.5 mo
Cloughesy et al. [45]	2016	NCT01156584	I	54	Recurrent GBM	N/A	(1) Toca 511 + Toca FC (2) External control	(1) mOS: 13.6 mo (2) mOS: 7.1 mo	N/A
Fenstermaker et al. [46]	2016	NCT01250470	I	9	Recurrent GBM SURVIVIN+	N/A	SVN53-67/M57-KLH (SurVaxM): conjugated survivin peptide mimic vaccine with KLH	mOS: 86.6 wk, OS-1 y: 77.8%	mPFS: 17.6 wk
Oji et al. [47]	2016	UMIN000002001	II	50	GBM	N/A	WT1-235 peptide vaccination	OS significantly prolonged (*p* = 0.001)	PFS significantly prolonged (*p* = 0.028)
Wheeler et al. [48]	2016	NCT00589875	II	182	Newly diagnosed GBM	36	(1) GMCI + SOC (2) SOC	(1) OS-1 y/2 y/3 y: 67%/35%/19% (2) OS-1 y/2 y/3 y: 57%/22%/8%	N/A
Alonso et al. [49]	2017	NCT01956734	I	61	Recurrent GBM	N/A	DNX-2401 + TMZ	OS-9 mo: 100%	N/A
O’Rourke et al. [50]	2017	NCT02209376	I	10	Recurrent GBM EGFRvIII+	N/A	CAR T-EGFRvIII+	mOS: 251 d	N/A
Kong et al. [51]	2017	NCT00807027	III	180	Newly diagnosed GBM	N/A	(1) ACT w/expansion of autologous CIK (cytokine-induced killer cells) + SOC (2) SOC	(1) mOS: 22.5 mo, OS-12 mo/18 mo/24 mo: 78.2%/57.2%/38.2% (2) mOS: 16.9 mo, OS-12 mo/18 mo/24 mo: 75.2%/45.1%/38.5%	(1) mPFS: 8.1 mo, PFS-12 mo/18 mo/24 mo: 28.3%/25.6%/18.4% (2) mPFS: 5.4 mo, PFS-12 mo/18 mo/24 mo: 22.6%/21.2%/13.4%
Ursu et al. [52]	2017	N/A	II	81	GBM	N/A	(1) CpG-ODN (administrated locally around the surgical cavity) + SOC (2) SOC	(1) OS-2 y: 31% (2) OS-2 y: 26%	(1) mPFS: 9 mo (2) mPFS: 8.5 mo
Inogés et al. [53]	2017	NCT01006044	II	31	Newly diagnosed GBM	N/A	Autologous whole tumor lysate-pulsed DCV + RT and CHT	mOS: 23.4 mo	mPFS: 12.7 mo
Geletneky et al. [54]	2017	NCT01301430	I/II	18	Progressive primary or recurrent GBM	6	H-1 PV (H-1 parvovirus)	mOS: 15.5 mo	PFS: 4 mo
Weller et al. [55]	2017	NCT01480479	III	745	Newly diagnosed GBM EGFRvIII+	12	(1) Rindopepimut (with KLH) + GM-CSF and TMZ (2) KLH and TMZ	(1) mOS: 20.1 mo (2) mOS: 20 mo	N/A
Zadeh et al. [56]	2018	NCT02798406	II	49	Recurrent GBM	N/A	DNX-2401, pembrolizumab	OS-9 mo: 100%	N/A
Peereboom et al. [57]	2018	NCT02078648	I/II	74	Recurrent GBM HLA-A2+	N/A	SL701/GM-CSF + poly-ICLC and bevacizumab	OS-12: 37%	
Cloughesy et al. [58]	2018	NCT01470794	I	56	Recurrent GBM	36	Toca 511 + Toca FC	mOS: 11.9 mo (95% CI, 10.7 mo to 15.1 mo)	N/A
Fried et al. [59]	2018	N/A	I	9	Pediatric DIPG	N/A	Pidilizumab (MDV9300) + RT	mOS: 15.6 mo	mPFS: 9.3 mo
Pellegatta et al. [60]	2018	N/A	II	24	GBM	17	Autologous tumor lysate-pulsed DCV + TMZ	mOS: 20.1, OS-1 y/2 y: 75%/37%	mPFS: 10.5 mo, PFS-6 mo: 79%, PFS-12 mo: 37.5%
Yao et al. [61]	2018	N/A	II	47	GBM	N/A	Autologous tumor lysate-pulsed DCV vs. placebo	OS significantly prolonged (*p* < 0.01)	PFS significantly prolonged (*p* = 0.03)
Wick et al. [62]	2018	NCT02149225	I	16	Newly diagnosed GBM HLA-A*02:01 or HLA-A*24:02+	N/A	APVAC1 or APVAC2 (multi-peptide vaccines)/GM-CSF + poly-ICLC + TMZ	mOS: 29 mo	mPFS: 14.2 mo
Desjardins et al. [63]	2018	NCT01491893	I	61	Recurrent supratentorial GBM	N/A	PVSRIPO (compared with an historical control group (n = 104) treated with standard therapy)	OS-6 mo/12 mo/24 mo/36 mo/48 mo/60 mo: 90%/54%/21%/21%/21%/21%	N/A
Buchroithner et al. [64]	2018	N/A	II	76	Newly diagnosed GBM	N/A	(1) Tumor lysate-charged autologous DCV (Audencel) + SOC (2) SOC	(1) mOS: 564 d (2) mOS: 568 d	(1) PFS-12 mo: 28.4% (2) PFS-12 mo: 24.5%
Bota et al. [65]	2018	N/A	II	9	Recurrent GBM	N/A	(1) Gliovac (ERC 1671) vaccine + bevacizumab (2) Placebo + bevacizumab	(1) mOS: 12.1 mo, OS-12 mo: 50% (2) mOS: 7.6 mo, OS-12 mo: 26%	(1) mPFS: 7.3 mo (2) mPFS: 5.4 mo
Lang et al. [66]	2018	NCT00805376	I	37	Recurrent HGG	N/A	(1) DNX-2401 intratecal injection (2) DNX-2401 intrathecal infusion + resection	(1) mOS: 9.5 mo (2) mOS: 13.0 mo	N/A
Kieran et al. [67]	2019	NCT00634231	I	12	GBM	N/A	AdV-tk plus valacyclovir + RT	OS: 24 mo	PFS: 37.3 and 47.7 mo
Todo et al. [68]	2019	UMIN000015995	II	30	Recurrent GBM	N/A	G47 delta (compared with an historical control group treated with standard therapy)	OS-12: 84.4%	N/A
Wen et al. [69]	2019	NCT01280552	II	124	Newly diagnosed GBM	40	(1) ICT-107 (peptide-pulsed DC vaccine) (2) Un-pulsed DCs	(1) mOS: 17.0 mo (2) mOS: 15.0 mo (HR = 0.87, *p* = 0.58)	N/A
Chiocca et al. [70]	2019	NCT02026271	I	31	Recurrent HGG	13	hIL-12 vector (Ad–RTS–hIL–12) injected in resection cavity + Veledimex (VDX, oral activator for hIL-12)	mOS in VDX 20 mg cohort: 12.7 mo, OS-12 mo/18 mo/24 mo in VDX 20 mg cohort: 60%/26.7%/13.3%, OS-12 mo in 10 mg/ 20 mg/ 30 mg/ 40 mg cohorts: 0%/60%/0%/30%	N/A
Cloughesy et al. [71]	2019	NCT02414165	II/III	403	Recurrent GBM	23	Toca 511 + Toca FC	mOS: 11.1 mo	N/A
Migliorini et al. [72]	2019	NCT01920191	I/II	19	Newly diagnosed GBM	N/A	RCHT + IMA950 multipeptide vaccine (w/ adjuvant poly-ICLC)	mOS: 10 mo (all patients) and 9.5 mo (GBM patients)	mPFS: 10 mo (all pt.) and 9.5 (GBM patients), PFS-6 mo/9 mo: 84% (all patients) and 63% (GBM patients)
Eoli et al. [73]	2019	NCT04002804	I/II	20	Recurrent GBM	9	(1) Autologous tumor lysate-pulsed DCV + TMZ (2) Autologous tumor lysate-pulsed DCV + TT preconditioning of the vaccine site	(1) OS-9: 33% (2) OS-9: 62.5%	N/A
Goff et al. [74]	2019	NCT01454596	I	18	Recurrent GBM EGFRvIII+	N/A	Autologous EGFRvIII-specific CAR T cells	mOS: 6.9 mo	mPFS: 1.3 mo
Cloughesy et al. [75]	2019	N/A	I	32	Recurrent GBM	16	(1) Neoadjuvant pembrolizumab + adjuvant pembrolizumab (2) Adjuvant pembrolizumab	N/A	(1) mPFS: 99.5 d (2) mPFS: 72.5 d
Cloughesy et al. [76]	2020	NCT02511405	III	256	Recurrent GBM	N/A	(1) VB-111, bevacizumab (2) Bevacizumab	(1) mOS: 6.8 mo (2) mOS: 7.9 mo	N/A
Mueller et al. [77]	2020	NCT02960230	I	29	New GBM with H3.3 K27M mutation	18	(1) H3.3 K27M peptide vaccine (2) H3.3 K27M peptide vaccine + Nivolumab	(1) mOS: 16.1 mo (2) mOS: 9.8 mo	N/A
Mishinov et al. [78]	2020	N/A	I	58	GBM	N/A	(1) Autologous tumor lysate-pulsed DCV + standard treatment (2) Allogeneic pooled lysates from more tumors—pulsed DCV + standard treatment (3) Maximum safe tumor resection + RT + CHT	(1) mOS: 16 mo (2) mOS: 15 mo (3) mOS: 14.5 mo	N/A
Awada et al. [79]	2020	NCT03291314	II	54	Recurrent GBM	25	(1) Axitinib + avelumab (2) Axitinib	(1) mOS: 26.6 wk (2) mOS: 18 wk	(1) PFS-6 mo: 22.2% (2) PFS-6 mo: 18.5%
Smith et al. [80]	2020	ACTRN12615000656538	I	25	Newly diagnosed GBM	12	ACT with CMV-specific autologous cytotoxic T cells	mOS: 21 mo, OS-6 mo/1 y/2 y: 92%/84%/36%	mPFS: 10 mo, PFS-6 mo/1 y/2 y: 72%/28%/16%
Reardon et al. [81]	2020	NCT02017717	III	369	Recurrent GBM	10	(1) Nivolumab (2) Bevacizumab	(1) mOS: 9.8 mo OS-6 mo/12 mo/18 mo:72.3%/42%/21% (2) mOS: 10 mo, OS-6 mo/12 mo/18 mo:78.2%/42%/21%	(1) mPFS: 1.5 mo, PFS-6 mo/12 mo/18 mo:15.7%/10.5%/5.8% (2) PFS-6 mo/12 mo/18 mo:29.6%/17.4%/8.9%
Reardon et al. [82]	2020	NCT01498328	II	73	Recurrent GBM EGFRvIII+	N/A	(1) Rindopepimut (with KLH) + GM-CSF and bevacizumab (2) KLH and bevacizumab	(1) PFS-6 mo: 28%, OS-24 mo: 20% (2) PFS-6 mo: 16%, OS-24 mo: 3%	(1) OS-24 mo: 20% (2) OS-24 mo: 3%
Mitsuya et al. [83]	2020	NTC0190103	II	16	Newly diagnosed GBM	72	5 synthetic peptides-pulsed DCV	mOS: 19 mo	N/A
Weathers et al. [84]	2020	NCT02661282	I/II	20	GBM	12	ACT with CMV-specific autologous cytotoxic T cells	OS-1 y: 50%, mOS: 12 mo	PFS-6 mo: 19%, mPFS: 1.3 mo
Yuce Sari et al. [85]	2021	N/A	I	8	Recurrent GBM	21	FSRT + neoadjuvant, concomitant, adjuvant nivolumab	mOS: 21.3 mo (from diagnosis) and 12.6 mo (from progression), OS-1 y/2 y: 88%/33% (from diagnosis) and 73%/0% (from progression)	mPFS: 2.3 mo
Duerinck et al. [86]	2021	NCT03233152	I	27	Recurrent GBM	22	IC (in the brain tissue lining the resection cavity) nivolumab and ipilimumab + intravenous nivolumab (compared with an historical control group (n = 469) treated with standard therapy)	mOS: 38 wk, OS-6 mo/1 y/2 y: 74.1%/40.7%/27%	N/A
Lim et al. [87]	2021	KCT0003815	I/II	14	Recurrent GBM	24	ACT (adoptive immune cell therapy) with activated NK cells and T lymphocytes from PBMC	mOS: 22. mo, OS-2 y: 35.7%	mPF6: 10 mo
Jacques et al. [88]	2021	NCT03047473	II	30	Newly diagnosed GBM	42	Avelumab (ICI) + SOC	mOS: 15.3 mo	mPFS: 9.7 mo
Jiang et al. [89]	2021	NCT03392545	I	30	Recurrent GBM and DMG (H3K27M-mutant)	23	Cyclophosphamide (Treg depletion) IC, immunoadjuvant Poly I:C + systemic immunoadjuvant Poly I:C and GM-CSF, low-dose re-irradiation	mOS: 362 d	mPFS: 88 d
Werlenius et al. [90]	2021	NCT02799238	II	62	Newly diagnosed GBM	N/A	(1) ALECSAT + SOC (2) SOC	(1) mOS: 19.2 mo (2) mOS: 18.3 mo	(1) mPFS: 7.8 mo (2) mPFS: 7.9 mo
Reardon et al. [91]	2021	NCT02054806	I	26	Recurrent GBM PD-L1+	14	Pembrolizumab	mOS: 13.1 mo, OS-6 mo/1 y/2 y: 75.8%/58%/31%	mPFS: 2.8 mo, PFS-6 mo/1 y/2 y: 37.7%/16.8%/8.4%
Nayak et al. [92]	2021	NCT02337491	II	80	Recurrent GBM	49	(1) Pembrolizumab + bevacizumab (2) Pembrolizumab	(1) mOS: 8.8 mo, OS-6 mo/12 mo/18 mo: 79.7%/44.3%/16.9% (2) mOS: 10.3 mo, OS-6 mo/12 mo/18 mo: 70%/30%/23.3%	(1) mPFS: 4.1 mo, PFS-6 mo/12 mo/18 mo: 26%/14%/10% (2) mPFS: 1.4 mo, PFS-6 mo/12 mo/18 mo: 6.7%/6.7%/3.3%
Sahebjam et al. [93]	2021	NCT02313272	I	32	Recurrent GBM	3	Pembrolizumab + RT + bevacizumab	(1) Bevacizumab-naïve: mOS: 13.4 mo, OS-6 mo/1 y/2 y: 91.7%/58.3%/16.7% (2) Bevacizumab-resistant: mOS: 9.3 mo, OS-6 mo/1 y: 87.5%/25%	(1) Bevacizumab-naïve: mPFS: 7.9 mo, PFS-6 mo/1 y: 66.7%/29.2% (2) Bevacizumab-resistant: mPFS: 6,5 mo, mOS: 9.3 mo, PFS-6 mo/1 y: 87.5%/25%
Bota et al. [94]	2022	N/A	N/A	21	Recurrent GBM	N/A	Bevacizumab + pembrolizumab or nivolumab + ERC 1671 vaccine	mOS: 19.63 mo, mOS-6 mo/1 y/2 y: 90.5%/61.1%/45.3%	mPFS: 9.14 mo, PFS-6 mo/1 y/2 y: 76.2%/47.62%/21.4%
Bota et al. [95]	2022	NCT03400917	II	57	GBM	N/A	Autologous tumor lysate-pulsed DCV + GM-CSF	mOS: 14 mo, OS-6 mo/12 mo/18 mo/24 mo: 87.5%/55.4%/38.5%/25.2%	mPFS: 8.5 mo, PFS-6 mo/12 mo/18 mo/24 mo: 69.7%/26.8%/16.1%/10%
Hu et al. [96]	2022	NCT02010606	I	36	GBM	N/A	Autologous tumor lysate-pulsed DCV + SOC	mOS: 20.36 mo	mPFS: 8.75 mo
Sampson et al. [97]	2022	NCT02858895	II	44	Recurrent GBM	N/A	MDNA55 (IL4 R targeting toxin) administered intratumorally using convection-enhanced delivery	(1) mOS: 11.6 mo, OS-1 y: 46% (2) Subgroup (n = 32) of IL4 R high and low patients treated with high-dose MDNA55]: mOS: 15 mo, OS-1 y: 55%	N/A
Omuro et al. [98]	2022	NCT02017717	I	117	Newly diagnosed GBM	N/A	(1) PART A: COHORT 1 c (n = 31) nivolumab + RT + TMZ; COHORT 1 d (n = 30) nivolumab + RT (2) PART B: COHORT 1 c (n = 28) nivolumab + RT + TMZ; COHORT 1 d (n = 28) nivolumab + RT	(1) PART A: COHORT 1 c mOS: 22 mo, COHORT 1 d mOS: 14.4 mo (2) PART B: COHORT 1 c mOS: 15 mo, COHORT 1 d mOS: 14 mo	(1) PART A: COHORT 1 c mPFS: 10 mo, COHORT 1 d mPFS: 5.6 mo (2) PART B: COHORT 1 c mPFS: 6.4 mo, COHORT 1 d mPFS: 6 mo
Lim et al. [99]	2022	NCT02667587	III	716	Newly diagnosed GBM with UNmethylated MGMT promoter	N/A	(1) Nivolumab + SOC (2) Placebo + SOC	(1) mOS: 28.9 mo (2) mOS: 32.1 mo	(1) mPFS: 10.6 mo (2) mPFS: 10.3 mo
Parney et al. [100]	2022	NCT01957956	I	20	Newly diagnosed GBM	35	Allogenetic GBM lysate-pulsed (mature) DCV + TMZ	mOS: 19 mo, OS-2 y/4 y: 25%/10%	mPFS: 9.7 mo
Chiocca et al. [101]	2022	NCT03636477	I	21	Recurrent GBM	N/A	Nivolumab + peritumoral injection of hIL-12 vector (Ad–RTS–hIL–12) + VDX	mOS all patients: 9.8 mo, mOS in VDX 10 mg: 16.9 mo, mOS in VDX 20 mg: 8.5 mo	N/A
Ogino et al. [102]	2022	NCT02549833	I	17	GBM	21	GBM6-AD + Poly-ICLC adjuvant: (1) neoadjuvant vaccination + surgery + adjuvant vaccination (2) surgery + adjuvant vaccination	N/A	No significant differences between PFS between two arms
Muragaki et al. [103]	2023	UMIN000010602	II	57	Newly diagnosed supratentorial GBM	N/A	(1) Autologous formalin-fixed GBM tumor vaccine (AFTV) + immune adjuvants (2) Identical placebo without fixed tumor tissue	(1) mOS: 25.6 mo, OS-3 y: 38%, PFS-3 y: 81%, OS-3 y: 80% (2) mOS: 31 mo, OS-3 y: 41%, PFS-3 y: 46%, OS-3 y: 54%	N/A
Mahase et al. [104]	2023	N/A	I	21	Recurrent GBM	N/A	(1) ICI + SBRT (fractionated stereotactic radiosurgery) (2) ICI (Pembrolizumab and Nivolumab)	(1) mOS: 7 mo (2) mOS: 6 mo	(1) mPFS: 2.8 mo (2) mPFS: 1 mo
Liu et al. [105]	2023	NCT03170141	I	8	GBM GD2+	24	Autologous GD2-specific 4SCAR-T cells	mOS: 10 mo	N/A
Guo et al. [106]	2023	NCT01765088	III	199	Newly diagnosed HGG	66	(1) IFN-α + TMZ (2) TMZ	(1) mOS: 26.7 mo and 24.7 mo (subgroup with UNmet MGMT prom), OS-2 y/5 y: 57.4%/18.1% (2) mOS: 18.8 mo and 17.4 mo (subgroup with UNmet MGMT prom), OS-2 y/5 y: 37.3%/9.1%	(1) mPFS: 14.8 mo, PFS-2 y/5 y: 27.9%/9.6% (2) mPFS: 12.9 mo, PFS-2 y/5 y: 18.5%/4.8%
Liau et al. [107]	2023	NCT00045968	III	331	GBM	N/A	Autologous tumor lysate-pulsed DCV + SOC	mOS: 19.3 mo (nGBM) and 13.2 mo (rGBM), OS-48 mo/60 mo: 15.7%/13% (nGBM), OS-24 mo/30 mo: 20.7%/9.7% (rGBM)	N/A
Burge et al. [108]	2023	NCT03383978	I	9	Recurrent GBM IDH wild type HER2 +	N/A	IC injection (into the margin of surgical cavity) of HER2-targeted CAR-NK cells NK-92/5.28 z (ACT, adoptive cell therapy)	mOS: 31 wk	mPFS: 7 wk
Lepski et al. [109]	2023	N/A	I/II	37	Recurrent GMB	N/A	Allogenic DC vaccination for GBM	OS: 26.9 mo, OS-6 mo/12 mo/18 mo/24 mo: 61.3%/46.6%/34.9%/26%	N/A

Abbreviations: Ab = antibody, ACT = adoptive cellular therapy, AFTV = autologous formalin-fixed tumor vaccine, ATV = autologous tumor vaccine, ALECSAT = autologous lymphoid effector cells specific against tumor, CAR T = chimeric antigen receptor T cell, CHT = chemotherapy, CpG-ODN = oligodeoxynucleotides containing unmethylated cytosine–guanosine motif, d = day, DC = dendritic cell, DIPG = diffuse intrinsic pontine glioma, DMG = diffuse midline glioma, DCV = dendritic cell vaccine, EBRT = external beam radiotherapy, FC = fusion cell, FRT = fractionated radiotherapy, FSRT = fractionated stereotactic radiotherapy, GAAs = glioma-associated antigens, GBM = glioblastoma multiforme, GMCI = gene-mediated cytotoxic immunotherapy, HGG = high-grade glioma, HSPPC-96 = heat-shock protein 96—peptide complex, HUVEC = human umbilical vein endothelial cell, IC = intracranial, ICIs = immune checkpoint inhibitors, mo = month, nGBM = newly diagnosed glioblastoma multiforme, N/A = not applicable, NDV = Newcastle disease virus, OS = overall survival, PBMC = peripheral blood mononuclear cell, PD = progressive disease, PFS = progression free survival, rGBM = recurrent glioblastoma multiforme, RCHT = radiochemotherapy, RIT = radioimmunotherapy, RT = radiotherapy, SOC = standard of care treatment (RT + TMZ), TMZ = temozolomide, TT = tetanus toxoid, VDX = veledimex, wk = week, y = year.

**Table 2:** Summary of the ongoing studies on GBM immunotherapies testing ICIs.

Trial Name	Phase	Patients (N)	Treatment	Outcomes
NCT02311920	I	32	Ipilimumab and/or nivolumab in combination with temozolomide	PFS and OS
NCT02336165	II	159	Durvalumab monotherapy, with bevacizumab or with radiotherapy	OS and PFS
NCT02658981	I	63	Anti-LAG3 or urelumab alone in combination with nivolumab	MTD
NCT03673787	I/II	87	Atezolizumab in combination with ipatasertib	DLT
NCT03743662	II	94	Nivolumab with radiation therapy and bevacizumab	T-lymphocyte density and safety
NCT03961971	I	15	Anti-Tim-3 in combination with anti-PD-1 and stereotactic radiosurgery	Serious adverse events
NCT04145115	II	37	Ipilimumab and nivolumab	DLT
NCT04396860	II/III	485	Ipilimumab and nivolumab plus radiation therapy	Efficacy and safety
NCT04606316	I	60	Nivolumab in combination with ipilimumab and surgery	Tumor infiltrating

Abbreviations: DLT = dose-limiting toxicity, MTD = maximum tolerated dose, OS = overall survival, PFS = progression free survival.

**Table 3:** Summary of the ongoing studies on GBM immunotherapies testing CVs.

Trial Name	Phase	Patients (N)	Treatment	Outcomes
NCT02366728	II	100	CMV pp65 DC vaccine +111In-labeled DC vaccine + Td Toxoid + basiliximab	OS
NCT02465268	II	175	pp65-shLAMP DC dendritic cell vaccine with GM-CSF	OS
NCT02924038	I	30	IMA-950 (peptide vaccine comprising multiple GAAs) and poly-ICLC ± varlilumab (immunostimulatory antiCD27 antibody)	Safety and T-cell responses
NCT02960230	I	29	H3.3K27 M peptide vaccine plus Td and poly-ICLC	Safety and OS
NCT03018288	II	108	pembrolizumab ± HSPPC-96 vaccine	1-year OS
NCT03400917	II	55	AV-GBM-1 (autologous dendritic cells loaded with tumor associated antigens from a short-term cell culture of autologous tumor cells)	OS
NCT04116658	II	52	EO2401 peptide vaccine	Safety and tolerability

Abbreviations: GBM = glioblastoma multiforme, OS = overall survival.

**Table 4:** Summary of the ongoing studies on GBM immunotherapies testing OVs.

Trial Name	Phase	Patients (N)	Treatment	Outcomes
NCT01301430	II	18	Human mesenchymal stem cells TG6002 (modified vaccinia virus) and 5-FC	Safety and DLT
NCT01470794	I	58	DNX-2440 conditionally replication-competent adenovirus with O × 40 ligand (T-cell stimulator)	DLT
NCT02062827	I	36	DNX-2401 (Delta-24-RGD adenovirus) ± surgery	MTD
NCT02798406	II	48	DNX-2401 (Delta-24-RGD adenovirus) and i.v. pembrolizumab (anti-PD-1 antibody)	ORR by interval tumor size change
NCT02986178	II	62	G207 (modified oncolytic strain of HSV-1) single-dose inoculation	OS at 24 months
NCT03152318	I	108	PVSRIPO (genetically recombinant nonpathogenic poliovirus:rhinovirus chimera) ± lomustine	MTD
NCT03294486	II	78	Ad5-DNX-2401 (oncolytic adenovirus) in bone marrow	Progression at 6 months
NCT03714334	I	24	rQNestin 34.5 v.2 (oncolytic HSV-1) + cyclophosphamide	Treatment-related adverse events
NCT03896568	I	36	Ad-RTS-hIL-12 + veledimex	Safety, DLT, and rate of tumor
NCT00390299	I	40	M032 (modified strain of HSV-1) by intratumoral infusion	Toxicity and MTD

Abbreviations: DLT = dose-limiting toxicity, MTD = maximum tolerated dose, ORR = overall response rate.

**Table 5:** Summary of the ongoing studies on GBM immunotherapies testing CAR T cells.

Trial Name	Phase	Patients (N)	Treatment	Outcomes
NCT01109095	I	16	HER2 CMV-specific CAR T cells	DLT
NCT01454596	I/II	18	EGFRvIII-directed CAR T cells with cyclophosphamide, fludarabine and aldesleukin	AEs and PFS
NCT02208362	I	92	IL13 Rα2-targeted CAR T cells	AEs and DLT
NCT02209376	I	11	EGFRvIII-directed CAR T cells	AEs
NCT04003649	I	60	IL13 Rα2-targeted CAR T cells with or without nivolumab and ipilimumab	AEs, DLT, feasibility, and OS
NCT04077866	I/II	40	B7-H3-targeted CAR T cells with or without temozolomide	OS and PFS
NCT04385173	I	12	B7-H3-targeted CAR T cells with temozolomide	AEs, MTD, OS, and PFS
NCT04661384	I	30	IL13 Rα2-targeted CAR T cells	AEs and OS

Abbreviations: AEs = adverse events, CAR T = chimeric antigen receptor T cell, DLT = dose-limiting toxicity, MTD = maximum tolerated dose, OS = overall survival, PFS = progression free survival.

## Data Availability

Data are available from a publicly accessible repository.

## Abbreviations

AA = anaplastic astrocytoma, Ab = antibody, ACT = adoptive cellular therapy, AE = adverse event, AFTV = autologous formalin-fixed tumor vaccine, ATV = autologous tumor vaccine, ALECSAT = autologous lymphoid effector cells specific against tumor, CAR T = chimeric antigen receptor T cell, CHT = chemotherapy, CpG-ODN = oligodeoxynucleotide containing unmethylated cytosine-guanosine motif, CSC = cancer stem cell, CV = cancer vaccine, d = day, DC = dendritic cell, DIPG = diffuse intrinsic pontine glioma, DLT = dose-limiting toxicity, DMG = diffuse midline glioma, DCV = dendritic cell vaccine, EBRT = external beam radiotherapy, FC = fusion cell, FRT = fractionated radiotherapy, FSRT = fractionated stereotactic radiotherapy, GAAs = glioma-associated antigens, GBM = glioblastoma multiforme, GMCI = gene-mediated cytotoxic immunotherapy, GSC = glioma stem cell, HGG = high-grade glioma, HSPPC-96 = heat-shock protein 96—peptide complex, HUVEC = human umbilical vein endothelial cell, IC = intracranial, ICI = immune checkpoint inhibitors, mo = month, MTD = maximum tolerated dose, nGBM = newly diagnosed glioblastoma multiforme, N/A = not applicable, NDV = Newcastle disease virus, ORR = overall response rate, OS = overall survival, OV= oncolytic virus, PBMC = peripheral blood mononuclear cell, PD = progressive disease, PFS = progression free survival, rGBM = recurrent glioblastoma multiforme, RCHT = radiochemotherapy, RIT = radioimmunotherapy, RT = radiotherapy, SOC = standard of care treatment (RT + TMZ), TMZ = temozolomide, TT = tetanus toxoid, VDX = Veledimex, wk = week, y = year.
